# Cep70 overexpression stimulates pancreatic cancer by inducing centrosome abnormality and microtubule disorganization

**DOI:** 10.1038/srep21263

**Published:** 2016-02-19

**Authors:** Songbo Xie, Juan Qin, Shiyu Liu, Yijun Zhang, Jun Wang, Xingjuan Shi, Dengwen Li, Jun Zhou, Min Liu

**Affiliations:** 1 Institute of Biomedical Sciences, College of Life Sciences, Key Laboratory of Animal Resistance of Shandong Province, Key Laboratory of Molecular and Nano Probes of the Ministry of Education, Shandong Normal University, Jinan 250014, China; 2 State Key Laboratory of Medicinal Chemical Biology, College of Life Sciences, Nankai University, Tianjin 300071, China

## Abstract

The centrosome is crucial for mitotic fidelity, and centrosome aberrations are associated with genomic instability and tumorigenesis. The centrosomal protein Cep70 has been reported to play a role in various cellular activities. However, whether this protein is involved in pathological processes remains unknown. In this study, we demonstrate that Cep70 is highly expressed in pancreatic cancer tissues. Cep70 expression correlates with clinicopathological parameters of pancreatic cancer, including histological grade, pathological tumor node metastasis stage, lymph node metastasis, and carbohydrate antigen 19-9 level. Depletion of Cep70 significantly suppresses pancreatic cancer cell proliferation and promotes apoptotic cell death, and exogenous expression of Cep70 can rescue the above effects. Cep70 also stimulates colony formation in soft agar and enhances tumor growth in mice. Our data further show that ectopic expression of Cep70 in pancreatic cancer cells results in the mislocalization of centrosomal proteins, including γ-tubulin and pericentrin, and the formation of intracellular aggregates. In addition, Cep70 overexpression leads to microtubule disorganization and the formation of multipolar spindles during mitosis. Our study thus unravels a critical role for Cep70 in pancreatic cancer and suggests Cep70 as a potential biomarker and therapeutic target for this deadly disease.

Pancreatic cancer is one of the most deadly malignancies, resulting in about 7% of cancer-related deaths worldwide^1^. Owing to the rapid growth and metastasis and lack of early detection, the prognosis of pancreatic cancer is extremely poor^2^,^3^. It is estimated that less than 25% of pancreatic cancer patients can survive within a year, and the five-year survival is no more than 5%^4^,^5^. Currently, the first-line chemotherapeutic option for patients with advanced pancreatic cancer is gemcitabine alone or in combination with other agents^5^,^6^. However, limited effectiveness and side effects are serious issues and challenge the utility of chemotherapy in many patients. Although our understanding of the genetic and genomic alterations, as well as the key signaling pathways underlying pancreatic tumorigenesis and progression, has been greatly improved over the past decades, the diagnosis and therapy of this malignancy remain to be major challenges^7^. Therefore, additional attempts to unravel molecular mechanisms underlying this dreadful disease for early detection, prevention, and treatment are needed.

The centrosome, an important organelle serving as the main microtubule organizing center, is essential for mitotic fidelity^8^,^9^. Centrosomes undergo maturation and segregation during the cell cycle, and aberrations in centrosome maturation have been implicated in tumorigenesis^10^,^11^,^12^. For example, abnormal expression of LGALS3BP (lectin galactoside-binding soluble 3 binding protein), a centriole- and basal body-associated protein, results in centrosome aberration and the development of cancer^13^. Cep70, a protein initially identified in a proteomic analysis^14^, is located at the centrosome throughout the cell cycle by interacting with γ-tubulin^15^. Cep70 plays a critical role in the regulation of microtubule dynamics, mitotic spindle formation, cell migration, and ciliogenesis^15^,^16^,^17^,^18^,^19^. However, whether Cep70 is involved in pathological processes remains unknown. In this study, our data present the first evidence that ectopic expression of Cep70 promotes pancreatic tumorigenesis by inducing centrosome abnormality and microtubule disorganization.

## Results

### Cep70 expression is elevated in pancreatic cancer and correlates with clinicopathological parameters

**Figure 1:**
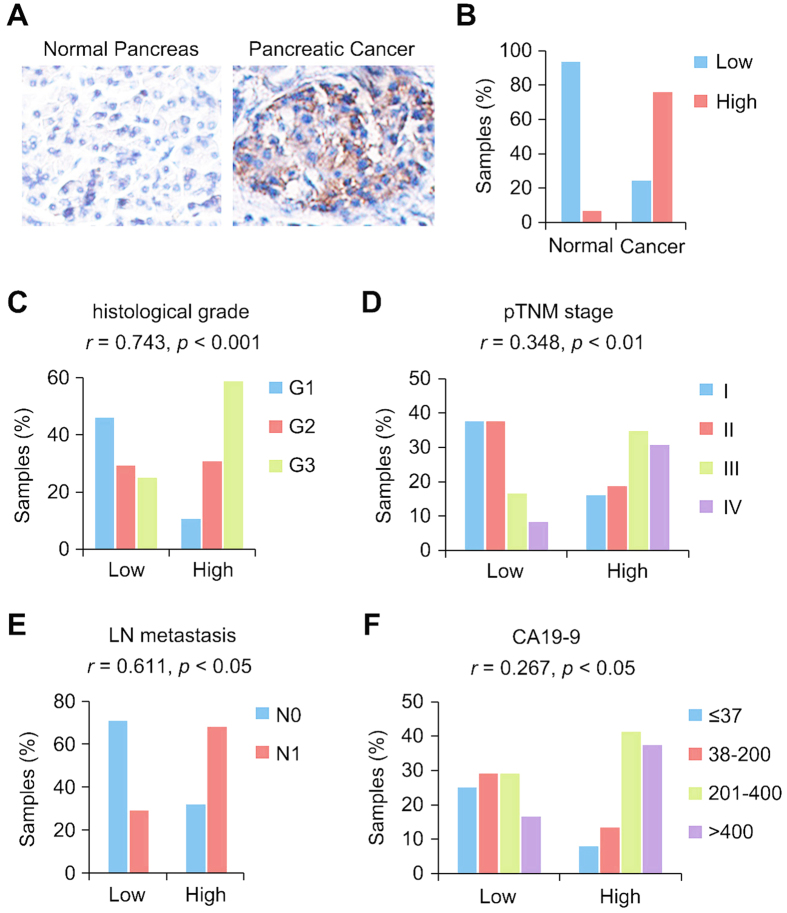
Cep70 is highly expressed in pancreatic cancer and correlates with clinicopathological parameters. (**A**) Representative images of immunohistochemical staining of Cep70 in normal pancreas and pancreatic cancer tissues. (**B**) Quantitative analysis of Cep70 expression in normal pancreas and pancreatic cancer tissues. (**C–F**) Correlation analyses between Cep70 expression and clinicopathological parameters of pancreatic cancer, including histological grade (**C**), pathological tumor node metastasis (pTNM) stage (**D**), lymph node (LN) metastasis (**E**), and carbohydrate antigen 19-9 (CA19-9) level (**F**). Correlation coefficient (*r*) and *p* values were calculated by the Spearman’s rank correlation test.

To dissect the role of Cep70 in pancreatic cancer, we first examined its expression in human pancreatic cancer tissues by immunohistochemical staining. Normal pancreatic tissues obtained from patients undergoing distal pancreatectomy for diseases other than pancreatic cancer were used as control. The expression of Cep70 in pancreatic cancer tissues was significantly up-regulated as compared with normal pancreatic tissues (Fig. 1A). Only 7.4% of normal pancreatic tissues showed high expression of Cep70, whereas 77.6% of pancreatic cancer tissues had high expression (Fig. 1B). Next, we investigated whether Cep70 expression correlates with the clinicopathological parameters of pancreatic cancer. We found that the level of Cep70 significantly correlated with histological grade (Fig. 1C), pathological tumor node metastasis (pTNM) stage (Fig. 1D), and lymph node (LN) metastasis (Fig. 1E). In addition, there was a correlation between the expression of Cep70 and the level of carbohydrate antigen 19-9 (CA19-9), the standard serum marker of pancreatic cancer (Fig. 1F). These data suggest that the up-regulation of Cep70 might be associated with the pathogenesis of pancreatic cancer.

### Ectopic expression of Cep70 in pancreatic cancer is independent of gene copy number gain

**Figure 2:**
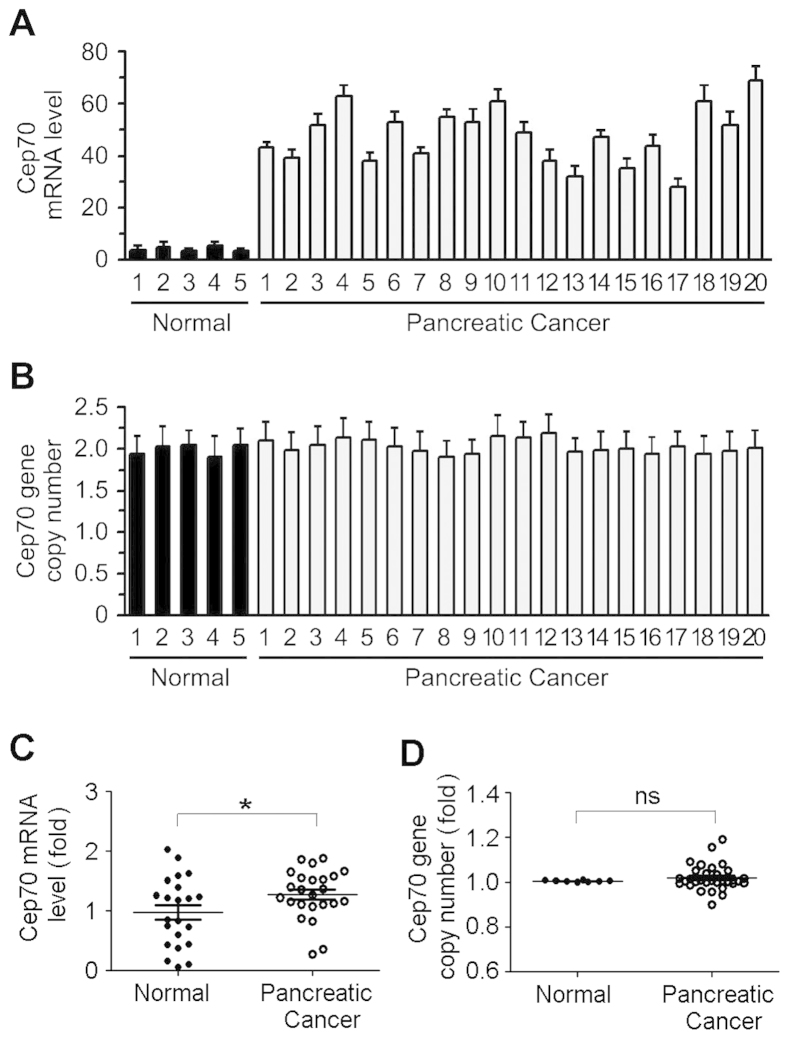
Determination of Cep70 mRNA level and gene copy number. (**A**) Quantitative real-time RT-PCR analysis of Cep70 mRNA expression in normal pancreas and pancreatic cancer tissues. (**B**) Quantitative real-time PCR analysis of Cep70 gene copy number in normal pancreas and pancreatic cancer tissues. (**C,D**) Quantification of Cep70 mRNA expression (**C**) and gene copy number (**D**) in normal pancreas and pancreatic cancer tissues, using the datasets in the Oncomine platform. *p < 0.05 versus normal; ns; not significant. Error bars indicate SEM.

To investigate the mechanism for elevated Cep70 expression in pancreatic cancer, we examined Cep70 mRNA level by quantitative real-time RT-PCR. Compared with normal pancreatic tissues, the level of Cep70 mRNA in pancreatic cancer tissues was remarkably increased, with an average of 12-fold increase (Fig. 2A). Since gene amplification frequently occurs in human pancreatic cancer^20^,^21^, we investigated whether Cep70 gene copy number is altered in pancreatic cancer tissues. By quantitative real-time PCR analysis, we found that Cep70 gene copy number in pancreatic cancer tissues was similar to that in normal pancreatic tissues (Fig. 2B). To verify the above results, we analyzed Cep70 mRNA expression and gene copy number using the datasets in the Oncomine platform. Consistent with our quantitative real-time RT-PCR results, the Ishikawa dataset showed that the level of Cep70 mRNA was significantly increased in pancreatic cancer tissues, as compared to normal pancreas (Fig. 2C). In addition, data from the TCGA database in the Oncomine platform showed that there was no significant difference in Cep70 gene copy number between normal pancreas and pancreatic cancer samples (Fig. 2D). Together, these results suggest that the aberrant expression of Cep70 in pancreatic cancer is independent of its gene copy number gain.

### Depletion of Cep70 suppresses pancreatic cancer cell proliferation and promotes apoptotic cell death

**Figure 3:**
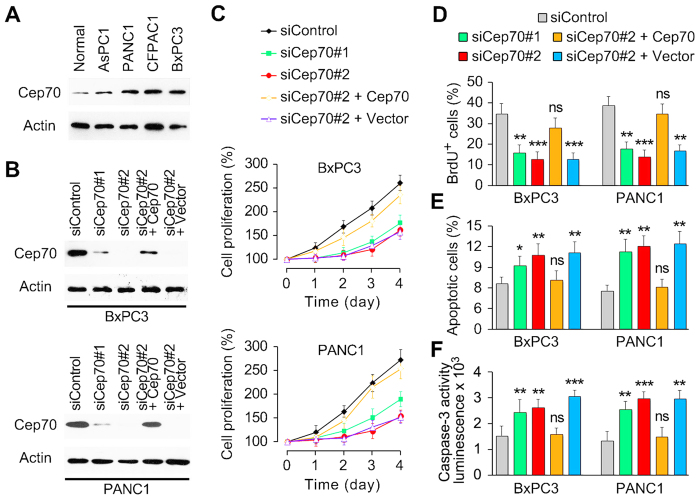
Loss of Cep70 suppresses pancreatic cancer cell proliferation and promotes apoptotic cell death. (**A**) Examination of Cep70 expression in normal pancreas and pancreatic cancer cell lines by immunoblotting. (**B**) Immunoblot analysis of Cep70 expression in BxPC3 and PANC1 cells transfected with the indicated siRNAs and plasmids. (C) Cells were transfected as indicated, and cell proliferation was determined by sulforhodamine B staining assay. (**D**) Effect of Cep70 on pancreatic cancer cell proliferation evaluated by BrdU incorporation assay. (**E**) Effect of Cep70 on apoptosis evaluated by staining cells with DAPI and examination of nuclear morphology. (**F**) Effect of Cep70 on apoptosis evaluated by analysis of caspase-3 activity with the luminogenic substrate Z-DEVD-aminoluciferin. *p < 0.05 versus siControl; **p < 0.01 versus siControl; ***p < 0.001 versus siControl; ns, not significant. Error bars indicate SEM.

To further examine the involvement of Cep70 in pancreatic cancer, we investigated its expression in four human pancreatic cancer cell lines, including AsPC1, PANC1, CFPAC1, and BxPC3. Consistent with the immunohistochemical results, Cep70 expression was up-regulated in all of these pancreatic cancer cell lines as compared to normal pancreas (Fig. 3A). We next studied the effect of Cep70 on pancreatic cancer cell proliferation. BxPC3 and PANC1 cells were treated with two different siRNAs targeting Cep70, one targeting its coding region and the other targeting its non-coding region. Both of these siRNAs could efficiently deplete the expression of Cep70 (Fig. 3B). By sulforhodamine B staining assay, which reflects the index of cell proliferation, we found that depletion of Cep70 markedly inhibited the proliferation of pancreatic cancer cells (Fig. 3C). In addition, exogenous expression of Cep70 could largely rescue the above effect in both BxPC3 and PANC1 cells (Fig. 3B,C).

To confirm the role of Cep70 in pancreatic cancer cell proliferation, we performed bromodeoxyuridine (BrdU) incorporation assay. siRNA-mediated depletion of Cep70 significantly attenuated the incorporation of BrdU during DNA replication, which was significantly restored by exogenous expression of Cep70 (Fig. 3D). We also investigated the effect of Cep70 on apoptotic cell death. By examination of nuclear morphology, we found that knockdown of Cep70 expression promoted apoptosis in BxPC3 and PANC1 cells, and this effect was rescued by exogenous expression of Cep70 (Fig. 3E). These results were confirmed by examination of caspase-3 activity with the luminogenic substrate Z-DEVD-aminoluciferin (Fig. 3F). Collectively, these data indicate that Cep70 promotes pancreatic cancer cell proliferation and inhibits apoptotic cell death.

### Cep70 promotes colony formation in soft agar and stimulates tumor growth in mice

**Figure 4:**
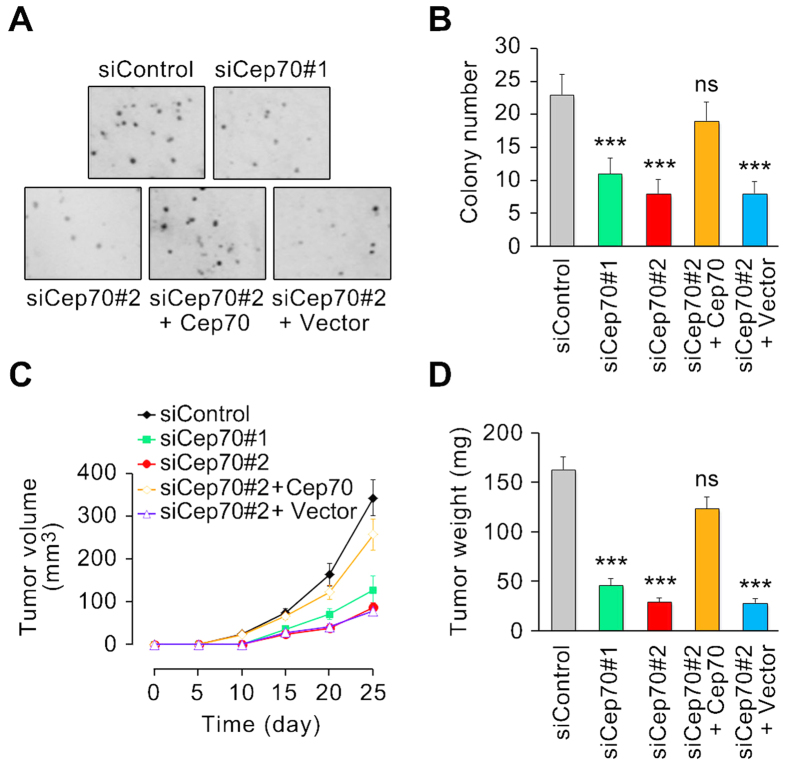
Cep70 promotes colony formation in soft agar and stimulates tumor growth in mice. (**A**) PANC1 cells were transfected with the indicated siRNAs and plasmids, and colony formation assay was performed in soft agar. (**B**) Experiments were performed as in panel A, and the number of colonies was counted. (**C**) PANC1 cells transfected with the indicated siRNAs and plasmids were injected into the flank of athymic nude mice, and the tumor volume was measured every 5 days. (**D**) Mice treated as in C were sacrificed 25 days post-injection, and tumors were isolated and weighed. ***p < 0.001 versus siControl; ns, not significant. Error bars indicate SEM.

The next question then is whether Cep70 plays a role in the development of pancreatic cancer. We first performed colony formation assay in soft agar to assess whether Cep70 is required for anchorage-independent growth of pancreatic cancer cells. We found that Cep70 siRNAs dramatically reduced the ability of pancreatic cancer cells to form colonies in soft agar, which was remarkably restored by exogenous expression of Cep70 (Fig. 4A,B). To substantiate the significance of Cep70 in pancreatic tumorigenesis, we altered the expression of Cep70 in pancreatic cancer cells and then injected these cells subcutaneously into the flank of athymic nude mice. We found that the tumor volume in Cep70 siRNA groups was much smaller than that in the control siRNA group (Fig. 4C). Notably, exogenous expression of Cep70 resulted in a substantial increase in tumor volume (Fig. 4C). We isolated tumors from mice 25 days post-injection and determined the tumor weight. As shown in Fig. 4D, Cep70 siRNAs dramatically reduced tumor weight, which was abolished by exogenous expression of Cep70. These results demonstrate a critical role for Cep70 in the development of pancreatic cancer.

### Cep70 overexpression results in centrosome abnormality

**Figure 5:**
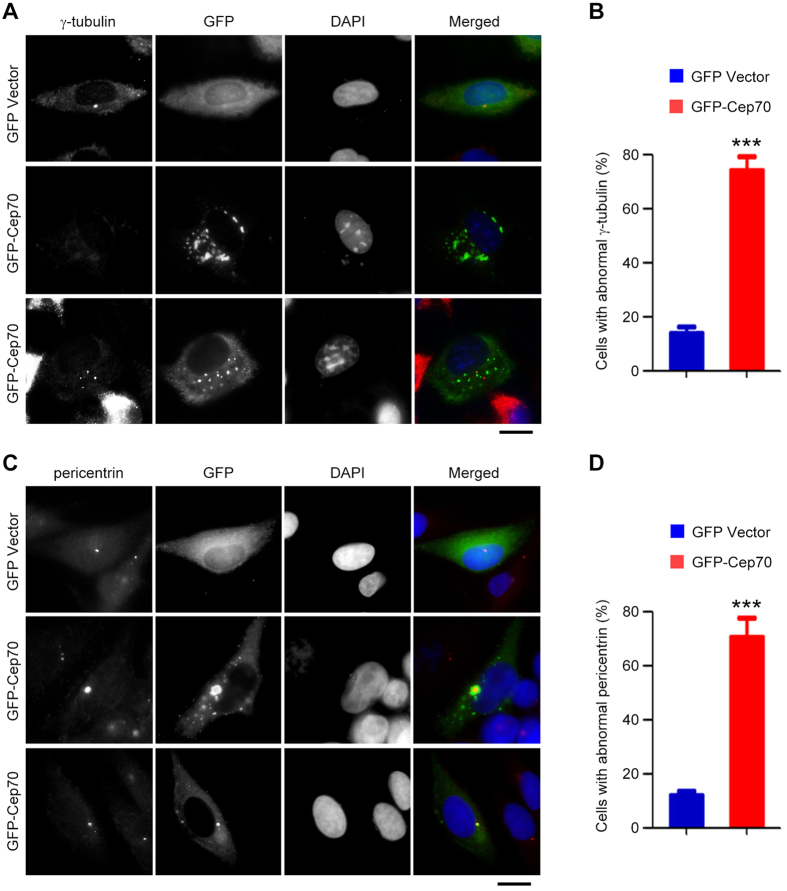
Cep70 overexpression causes centrosome abnormality in pancreatic cancer cells. (**A**) PANC1 cells transfected with GFP vector or GFP-Cep70 were immunostained with anti-γ-tubulin antibody and DAPI. (**B**) Experiments were performed as in A, and the percentage of cells with abnormal γ-tubulin localization was assessed. (**C**) PANC1 cells transfected with GFP vector or GFP-Cep70 were immunostained with anti-pericentrin antibody and DAPI. (**D**) Experiments were performed as in C, and the percentage of cells with abnormal pericentrin localization was assessed. ***p < 0.001 versus GFP vector. Error bars indicate SEM.

To understand the molecular mechanism by which ectopic expression of Cep70 promotes pancreatic tumorigenesis, we transfected GFP-Cep70 into pancreatic cancer cells and examined the localization of centrosomal proteins. Since Cep70 localization at the centrosome depends on its interaction with γ-tubulin, we first examined the effect of Cep70 overexpression on γ-tubulin localization in pancreatic cancer cells. Immunofluorescence microscopy revealed a typical centrosomal localization of γ-tubulin in control cells; by contrast, γ-tubulin was difficult to detect or formed multiple irregular aggregates in the majority of cells overexpressing GFP-Cep70 (Fig. 5A,B). To verify the effect of Cep70 on centrosome abnormality, we examined pericentrin, another centrosomal marker. We found that GFP-Cep70 overexpression resulted in aggresome-like or dispersed distribution of pericentrin (Fig. 5C,D). These data suggest that ectopic expression of Cep70 disrupts the localization of centrosomal components and causes centrosome abnormality.

### Cep70 overexpression causes microtubule disorganization and multipolar spindle formation

**Figure 6:**
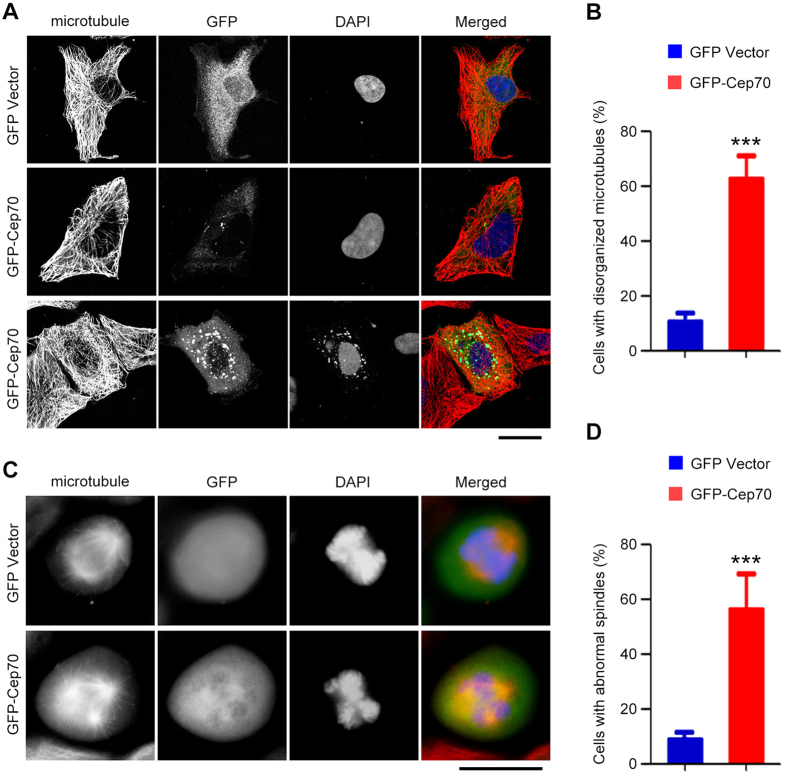
Cep70 overexpression results in microtubule disorganization and multipolar spindle formation. (**A**) PANC1 cells transfected with GFP vector or GFP-Cep70 were immunostained with anti-α-tubulin antibody and DAPI. (**B**) Experiments were performed as in A, and the percentage of cells with disorganized microtubules was assessed. (**C**) PANC1 cells transfected with GFP vector or GFP-Cep70 were immunostained with anti-α-tubulin antibody and DAPI, and mitotic cells were examined. (**D**) Experiments were performed as in C, and the percentage of mitotic cells with abnormal spindles was assessed. ***p < 0.001 versus GFP vector. Error bars indicate SEM.

Given the significance of the centrosome in microtubule organization, we analyzed the effect of Cep70 overexpression on microtubules. Classic radial microtubule arrays were observed in control pancreatic cancer cells; however, most of the GFP-Cep70 expressing cells exhibited disorganized microtubules (Fig. 6A,B). Since the centrosome plays a crucial role in bipolar spindle formation, we then studied the effect of Cep70 overexpression on the mitotic spindle in pancreatic cancer cells. By immunostaining, we found that control mitotic cells exhibited normal bipolar spindles; however, overexpression of Cep70 resulted in a substantial increase in the percentage of cells with multipolar spindles (Fig. 6C,D). Collectively, these data demonstrate that ectopic expression of Cep70 causes microtubule disorganization and multipolar spindle formation in pancreatic cancer cells.

## Discussion

The prognosis of pancreatic cancer is extremely poor, and there are few efficacious therapeutic options for this deadly disease^22^. A better understanding of the molecular mechanisms underlying pancreatic tumorigenesis and progression is thus in great demand. In this study, we reveal that the expression of Cep70 is elevated in pancreatic cancer tissues. Interestingly, Cep70 expression correlates with clininopathological parameters of pancreatic cancer. These findings indicate that Cep70 might be a potential biomarker and therapeutic target for pancreatic cancer. Despite the significant up-regulation of Cep70 mRNA expression, its gene copy number is not obviously changed in pancreatic cancer tissues, suggesting that Cep70 up-regulation in this disease is not due to gene amplification. Since numerous epigenetic alterations have been observed in pancreatic cancer and the prevalence grows as lesions become of more advanced stage^23^,^24^, it is possible that epigenetic modifications, such as promoter demethylation and histone modifications, might contribute to Cep70 up-regulation in pancreatic cancer.

Centrosome aberrations, including structural and functional aberrations, have been implicated in tumorigenesis. For example, overexpression of the centrosomal protein Nlp (ninein-like protein) causes the formation of intracellular aggregates around the centrosome, leading to the impairment of mitotic spindle formation^25^. In addition, the LIM (Lin11, Isl-1, and Mec-3) domain-containing protein LMO4 (LIM-only protein 4) is highly expressed in breast cancer, and its aberrant expression leads to centrosome amplification and defects in spindle formation^26^. Similarly, depletion of the p53 tumor suppressor produces supernumerary centrosomes, thereby causing unequal segregation of chromosomes and genetic instability^27^. The present study demonstrates a critical role for Cep70 in pancreatic cancer cell proliferation and tumor growth in mice. Our data also show that Cep70 overexpression in pancreatic cancer cells results in the mislocalization of γ-tubulin and pericentrin and formation of protein aggregates. Given the vital role of the centrosome in bipolar spindle formation and subsequent chromosome segregation^28^,^29^, it is possible that the impaired spindle formation by ectopic expression of Cep70 may trigger chromosomal instability.

At present, the precise molecular mechanisms of how Cep70 overexpression leads to centrosome abnormality in pancreatic cancer cells remain elusive. It has been shown previously that the cylindromatosis (CYLD) tumor suppressor deubiquitinates Cep70 and promotes its centrosomal localization, thereby contributing to ciliogenesis^19^. Loss of CYLD disassociates Cep70 from the centrosome, resulting in disorganization of basal bodies and axenomes and defects in ciliogenesis^19^. Our data reveal that ectopically expressed Cep70 forms multiple intracellular aggregates. It is possible that overexpression of Cep70 impairs its physical or functional interactions with other centrosomal proteins, such as γ-tubulin and pericentrin. In this scenario, it will be interesting to investigate in the future the effect of Cep70 overexpression on the molecular architecture and function of the centrosome, especially in the setting of pancreatic cancer pathogenesis.

## Methods

### Materials

Sulforhodamine B, BrdU, and 4′,6-diamidino-2-phenylindole (DAPI) were purchased from Sigma-Aldrich. Antibodies against β-actin, α-tubulin, γ-tubulin , and BrdU (Sigma-Aldrich) and pericentrin (Covance) were obtained from the indicated sources. Cep70 antibody was generated as described previously^15^. Horseradish peroxidase-conjugated secondary antibodies were purchased from Santa Cruz Biotechnology. Rhodamine-conjugated secondary antibody was from Jackson ImmunoResearch Laboratories. GFP-Cep70 plasmid, Cep70 siRNAs, and luciferase control siRNA were described previously^16^.

### Cell culture and transfection

AsPC1, PANC1, CFPAC1, and BxPC3 human pancreatic cancer cell lines were purchased from the American Type Culture Collection. AsPC1 and BxPC3 cells were cultured in RPMI-1640 medium supplemented with 10% fetal bovine serum (FBS). PANC1 cells were cultured in Dulbecco’s Modified Eagle’s Medium supplemented with 10% FBS. CFPAC1 cells were cultured in Iscove’s Modified Dulbecco’s Medium supplemented with 10% FBS. Cells were cultured in 5% CO_2_ incubator at 37^o^ C. Plasmids and siRNAs were transfected into cells by using TurboFect (Thermo Scientific) and DharmaFECT1 (Dharmacon), respectively.

### Human tissues, mice, and ethical declaration

Human pancreatic cancer tissues were obtained from patients who underwent surgical resection at Shanxian Dongda Hospital, and human normal pancreatic tissues were obtained from patients who underwent distal pancreatectomy at Shanxian Dongda Hospital for diseases other than pancreatic cancer. Cancer tissues were stratified into different subgroups based on grading, staging, lymph node metastasis, and preoperative CA19-9 cutoff values ( ≤37 U/ml refers to normal, >37 U/ml refers to positive, >200 U/ml refers to an association with poor prognosis, and > 400 U/ml refers to an association with worse prognosis). Written informed consent was obtained from patients in the study. Use of human tissues in this study was approved by the Ethics Committee of Nankai University. Use of mice in this study was approved by the Animal Care and Use Committee of Nankai University. All human and mouse experiments were carried out in accordance with the approved guidelines.

### Analysis of Oncomine platform data

By searching Cep70 in the Oncomine platform and inputting the filters, Cep70 mRNA expression and DNA copy number datasets were obtained. By sorting the data based on the *p* value, Ishikawa pancreatic cancer database for mRNA expression analysis^30^ and TCGA pancreatic cancer database for DNA copy number analysis were selected. The log2 median-centered intensity data were transformed and dot figures were plotted by using the GraphPad software.

### Sulforhodamine B staining and BrdU incorporation

BxPC3 and PANC1 cells transfected with siRNAs and/or plasmids were seeded in 96-well plates. Cells were fixed and stained with sulforhodamine B as described previously^31^,^32^. For BrdU incorporation assay, cells grown on glass coverslips were treated with 10 μM BrdU for 45 minutes and then fixed with 70% ethanol. After denaturation of cellular DNA with HCl, cells were probed with anti-BrdU antibody and rhodamine-conjugated secondary antibody. The percentage of BrdU-positive cells was analyzed by fluorescence microscopy.

### Quantitative real-time RT-PCR analysis

Total RNA was prepared from normal pancreas and pancreatic adenocarcinomas using the Trizol reagent according to manufacturer’s instructions (Invitrogen), and cDNA was prepared from the RNA using the Superscript kit (Invitrogen). Genomic DNA was isolated using a tissue DNA isolation kit (Qiagen). Quantitative real-time PCR was performed using the SYBR Premix Ex Taq reagent (Takara) as per the manufacturer’s instructions.

### Immunohistochemistry

Pancreatic cancer and normal pancreatic tissues were immunostained as described previously^21^,^33^. Briefly, sections were deparaffinized and rehydrated with xylene and graded alcohols. After antigen retrieval and inactivation of endogenous peroxidase, sections were incubated with goat serum, anti-Cep70 antibody, biotinylated secondary antibody, and streptavidin-biotin-peroxidase. Daminobenzidine was used as a chromogen substrate, and haematoxylin counterstaining was then performed. Cep70 level was determined by assessing both the intensity of staining (0 = negative; 1 = low; 2 = medium; 3 = high) and the percentage of stained cells (0 = 0% stained; 1 = 1–25% stained; 2 = 26–50% stained; 3 = 51–100% stained) as described previously^31^, and samples with a multiplied score (intensity score × percentage score) ≤ 3 were considered as low Cep70 expression and >3 as high Cep70 expression.

### Immunoblot analysis

Proteins were separated by sodium dodecyl sulfate-polyacrylamide gel electrophoresis. Proteins were then transferred onto polyvinylidene difluoride membranes (Millipore), followed by blocking with Tris-buffered saline containing 0.2% Tween 20 and 5% fat-free dry milk for 2 hours. Membranes were incubated with primary antibodies and then horseradish peroxidase-conjugated secondary antibodies as described^34^. Specific proteins were detected with enhanced chemiluminescence detection reagent (Pierce) according to the manufacturer’s protocol.

### Soft agar colony formation

Cells were mixed with 0.3% agar, followed by seeding onto a six-well plate containing 0.6% agar and incubated for 2 weeks. The colonies were then fixed with methanol and stained with 0.1% crystal violet. Photographs were taken and the number of colonies at each well was counted.

### Tumor growth in mice

Cells were injected subcutaneously into the right flanks of male athymic nude mice. Tumor volume was measured every 5 days with a vernier caliper and calculated as described previously^35^,^36^. The mice were sacrificed 25 days post-injection. Tumors were then isolated from mice, photographed, and weighed.

### Immunofluorescence microscopy

Immunofluorescence staining of pancreatic cancer cells was performed as described previously^37^,^38^. In brief, cells grown on glass coverslips were fixed with methanol at −20 °C for 5 minutes and then blocked with 2% bovine serine albumin in PBS for 20 minutes. Cells were incubated with primary antibodies and then with rhodamine-conjugated secondary antibodies. Nuclei were stained with DAPI. Coverslips were mounted with 90% glycerol in PBS and then examined with an Axio Observer A1 fluorescence microscope (Carl Zeiss, Inc.).

### Apoptosis assays

Cells grown on coverslips were stained with DAPI. The percentage of apoptotic cells was quantified by fluorescence microscopic analysis of nuclear morphology. Caspase-3 activity was examined by measuring the luminescence resulting from the cleavage of Z-DEVD-aminoluciferin (Promega) as described previously^39^.

### Statistics

Analysis of statistical significance was performed by the Student’s t-test for comparison between two groups and by the ANOVA test for multiple comparisons. Correlation coefficient was calculated by the Spearman’s rank correlation test.

## Additional Information

**How to cite this article**: Xie, S. *et al.* Cep70 overexpression stimulates pancreatic cancer by inducing centrosome abnormality and microtubule disorganization. *Sci. Rep.* **6**, 21263; doi: 10.1038/srep21263 (2016).
